# Green Synthesis of Zinc Oxide Nanoparticles Using Red Seaweed for the Elimination of Organic Toxic Dye from an Aqueous Solution

**DOI:** 10.3390/ma15155169

**Published:** 2022-07-26

**Authors:** Abdallah Tageldein Mansour, Ahmed E. Alprol, Mohamed Khedawy, Khamael M. Abualnaja, Tarek A. Shalaby, Gamal Rayan, Khaled M. A. Ramadan, Mohamed Ashour

**Affiliations:** 1 Animal and Fish Production Department, College of Agricultural and Food Sciences, King Faisal University, P.O. Box 420, Al-Ahsa 31982, Saudi Arabia; gahmed@kfu.edu.sa; 2 Fish and Animal Production Department, Faculty of Agriculture (Saba Basha), Alexandria University, Alexandria 21531, Egypt; 3 National Institute of Oceanography and Fisheries (NIOF), Cairo 11516, Egypt; dr_khedawy@yahoo.com; 4 Department of Chemistry, College of Science, Taif University, P.O. Box 11099, Taif 21944, Saudi Arabia; k.ala@tu.edu.sa; 5 Department of Arid Land Agriculture, College of Agricultural and Food Science, King Faisal University, P.O. Box 400, Al-Ahsa 31982, Saudi Arabia; tshalaby@kfu.edu.sa; 6 Horticulture Department, Faculty of Agriculture, Kafrelsheikh University, Kafr El-Sheikh 33516, Egypt; 7 Central Laboratories, Department of Chemistry, King Faisal University, P.O. Box 420, Al-Ahsa 31982, Saudi Arabia; kramadan@kfu.edu.sa; 8 Department of Biochemistry, Faculty of Agriculture, Ain Shams University, Cairo 11566, Egypt

**Keywords:** green ZnO-NPs, IV2R, equilibrium modeling, thermodynamic parameters, seaweed extract, adsorption process

## Abstract

This study aims to produce green zinc oxide nanoparticles (ZnO-NPs) derived from red seaweed (*Pterocladia Capillacea*) and evaluate their potential to absorb Ismate violet 2R (IV2R) ions from an aqueous solution. UV-vis spectrophotometry, Fourier-transform infrared spectroscopy (FTIR), scanning electron microscopy (SEM), X-ray diffraction (XRD), and a Brunauer–Emmett–Teller surface area analysis (BET) were used to analyze the structural, morphological, and optical features of the synthesized nanoparticles. The change in color of the chemical solution revealed the formation of zinc oxide nanoparticles. The FTIR examination confirmed the synthesis of both Zn and ZnO nanoparticle powder, with a BET surface area of 113.751 m^2^ g^−1^ and an average pore size of 2.527 nm for the synthesized adsorbent. Furthermore, the maximum removal effectiveness of IV2R was 99% when 0.08 g ZnO-NPs was applied at a pH of 6, a temperature of 55 °C, and a contact time of 120 min. The dye adsorption capacity of the ZnO-NPs was 72.24 mg g^−1^. The adsorption process was also controlled by the Freundlich adsorption model and pseudo-second-order reaction kinetics. The adsorption of IV2R ions onto the ZnO-NPs could be represented as a nonideal and reversible sorption process of a nonuniform surface, according to Freundlich adsorption isotherms. In addition, the constant values of the model parameters were determined using various nonlinear regression error functions. Moreover, thermodynamic parameters such as entropy change, enthalpy change, and free energy change were investigated; the adsorption process was spontaneous and endothermic. The high capacity of the ZnO-NPs synthesized by red seaweed promotes them as promising substances for applications in water treatment for the removal of IV2R dye from aqueous systems.

## 1. Introduction

Green nanotechnology is a methodology for creating nanomaterials that is devoid of or employs fewer toxic substances throughout the generation process [1]. Nanomaterial synthesis is one of the most challenging and rapidly growing areas of nanotechnology [2]. Metal nanoparticles have several applications in environmental pollution treatment, including heavy metals, degradation of toxic dyes, and antimicrobial substances [3]. Several researchers have presented numerous mechanistic ways to explain the hidden process behind the green synthesis of ZnO-NPs. As reported by Frattini et al. [4], NPs are generated both inside living algae and within sun-dried biomass. Nanomaterials are produced by reducing the amount of energy used throughout the production process [5]. In water or organic solvents, zinc ions are stable and dispersed colloidally. Nevertheless, zinc is a highly toxic chemical element, and zinc oxide has a more pronounced effect on microorganisms than on eukaryotic cells, as shown in the work of [6]. Nanoparticle synthesis can be achieved via physical, chemical, or biological means [7]. Although all chemical techniques are capable of producing clean, well-defined nanoparticles, they are costly and potentially hazardous to the environment. The various utilizations of biological biomasses, including yeast, bacteria, fungi, several plant extracts, and different algae extracts, are low-cost, environmentally friendly, and safe alternatives to existing nanoparticle production technologies [8]. The green synthesis of nanoparticles is used in the creation of highly stable nanoparticles.

Aquatic plants (microalgae and seaweeds) have the highest photosynthetic efficiency, are the highest biomass producers, are resistant to several pollutants, and have the ability to grow on land that is often inappropriate for other uses [9]. The cells of aquatic plants, such as seaweeds, have bioactive compounds and contain functional groups, including carboxyl, hydroxyl, amino, and sulphate, that can act as metal-binding sites between the adsorbent and adsorbate [10].

Algae nanoparticles are quite stable in solutions, eco-friendly, and safe to a large extent in several fields [9]. Furthermore, algal cells are sustainable resources that are extensively used as biomaterials for food [11], feed [5,12,13], fertilizers [14,15], bioenergy [16], and wastewater treatments [14,17]. Seaweeds have significant metal-binding capacities due to the presence of proteins, polysaccharides, and lipids on their cell wall surfaces that include several functional groups [18].

One of the most important environmental challenges, particularly in emerging countries, has been identified as irregular industrial development. The textile, plastic, and paper sectors all produce large amounts of wastewater containing synthetic dyes [19]. Synthetic dyes are utilized in a variety of industries, including textiles, leather, plastics, photography, medicine, cosmetics, and food [20,21]. Direct, reactive, acidic, and basic dyes are the most common types of dyes used in these industries [22]. Dye-containing wastewater has negative consequences for both the aquatic environment [23] and human health [24,25]. It is poisonous and can harm human health by causing damage to the neurological system, brain, eyes, liver, and skin [26]. These poisonous substances can endanger animals, plants, and humans. Dye removal from wastewater is accomplished using physical, chemical, and biological techniques. To remove colors from wastewater, a variety of processes are applied, including adsorption, advanced oxidation, membrane separation, and biological and electrochemical degradation [27].

Many companies utilize the adsorption process to eliminate harmful pollutants because it is one of the most important methods for water purification [1,28,29]. Adsorption is a viable approach because of its simple design, low cost, availability, and desire to eliminate color at high concentrations [30,31]. Adsorption is also a favored way of treating aqueous effluent in industrial operations for a variety of separation and purification applications. The sorption process is without a doubt the most essential physicochemical process for several organic and inorganic materials’ uptake in aquatic environments [32]. The extent of adsorption is affected by factors including pH, contact time, doses of substrates, adsorbing ion, temperature, and the presence of competing and complex ions [33]. 

Nanosized zinc oxides have a number of advantages, including strong surface reactivity and ease of synthesis from abundant natural materials, and they are a destructive sorbent compared to their commercial analogues. They are also confirmed to be excellent materials as adsorbents [34]. Moreover, ZnO-NPs have been selected as an adsorbent material because they have greater surface areas, more active surface sites than bulk materials, and excellent pollutant removal capacities [35,36,37]. In addition, ZnO-NPs as semiconductors have gained attention for their wide range of applications, including optoelectronics, optics, electronics, and dye removal employing environmentally benign synthesis components including fungi, bacteria, and marine macroalgae [38,39].

In the present study, a green synthesis of zinc oxide (ZnO) nanoparticles is created as an adsorbent biomaterial for the removal of Ismate violet 2R dye from an aqueous solution under varied circumstances by applying a batch adsorption technique. Additionally, ZnO is discovered to be more effective and, possibly, more beneficial than other metals for the biosynthesis of nanoparticles (NPs) for medical applications. Furthermore, the green synthesis of zinc oxide nanoparticles is characterized using UV-vis, SEM, FTIR, X-ray diffraction, and a PET surface area analysis. Additionally, the kinetics, thermodynamics, and equilibrium of IV2R removal from aqueous solutions applying this adsorbent are examined.

## 2. Materials and Methods

### 2.1. Collection and Preparation of Seaweeds

Seaweed (*Pterocladia Capillacea*) samples were collected from the Alexandria coast of Egypt. The harvested *P. Capillacea* biomass was washed once with both seawater and tap water and twice with distilled water to remove the exterior grit and salt [40]. The seaweed biomass was dried for 72 h at 70 °C, ground with a blender, and sieved to obtain particles that passed through a laboratory test sieve with a mesh size of 125 µm. The homogenous suspension of *P. Capillacea* biomass was filtered and dried at 70 °C for 72 h or until a consistent weight was achieved and then stored at room temperature.

### 2.2. ZnO Nanoparticle Synthesis

The dried *P. Capillacea* powder (2 g) was mixed with 100 mL double-distilled water, heated to 100 °C, and then filtered through filter paper. The production of green ZnO-NPs was applied according to a procedure described previously [41,42]. An amount of 50 mL aqueous extract of *P. Capillacea* was added to a 2 mM solution of Zn (Ac) 2H_2_O, followed by the drop-wise addition of 2 M NaOH until the pH reached 12. The mixture was stirred continuously at 65 °C for 2–3 h. The pale white solid product was collected using centrifugation at 5000 rpm for 10 min, followed by meticulous washing with distilled water and drying overnight at 80 °C.

### 2.3. Reagents and Chemicals

Table 1 shows the chemical structure and certain features of Ismate violet 2R (IV2R) dye. All the used reagents and chemicals were of analytical quality (Merck Company, Darmstadt, Germany). The IV2R dye was dissolved in 1 L double-distilled water to make a stock solution (1000 mg L^−1^). Dye solutions with concentrations in the range of 10–80 mg L^−1^ were generated for treatment studies by diluting the stock solution with double-distilled water.

### 2.4. Characterization

SEM was used to examine the morphology of the ZnO-NPs (SEM, Vega Tescan TS5136MM). Using KBr pellets, the FTIR spectra were recorded with a Nicolet 6700 FTIR spectrometric analyzer. Furthermore, the BET technique was used to compute the specific surface area in the relative pressure range of 0.05–0.3. The Barrett–Joyner–Halenda (BJH) method was used to compute the mesopore size and distribution from the desorption curves, whereas the t-plot approach was used to calculate the micropore area values. The porosity (using the DFT approach) and surface area were measured using a N_2_ adsorption–desorption isotherm analysis (Tristar 3000 apparatus, Micrometrics Instrument Corp., Norcross, GA, USA) (BET method). A UV-vis spectrophotometer was used to obtain the spectrophotometric readings (UV 4000, MRI, Stuttgart, Germany).

An XRD (D/Max 2550PC, Rigaku, Japan) diffractometer was used for X-ray diffractometry. The reflection-scanning mode was used to record the radical scan while changing 2 θ from 0° to 100°. A pH meter was used to determine the pH of the solution (Metrohm Herisau Digital E 532, Herisau, Switzerland).

### 2.5. Adsorption Equilibrium Procedure

#### 2.5.1. Batch Adsorption Studies

In a 100 mL conical flask, batch experimental trials for adsorption equilibrium studies were performed by mixing 50 mL IV2R solution with several concentrations of 10, 20, 40, 60, and 80 mg L^−1^. In the flasks, the adsorbent concentrations were 0.005, 0.01, 0.02, 0.04, and 0.8 g. The mixtures were shaken for 180 min at a steady speed of 200 rpm at an appropriate temperature. The temperatures used in the studies were 25, 30, 35, 45, and 55 °C. With 0.1 M HCl or 0.1 M NaOH, the pH values were altered to 2, 4, 6, 8, and 10. The mixing time was also examined (15, 30, 60, 120, and 180 min). The mixtures were filtered after the solutions reached equilibrium, and thus, the adsorbents were removed from the solutions. A UV-visible spectrophotometer set to maximum adsorption wavelength was used to determine the residual IV2R concentrations in the solutions (550 nm). Equation (1) was used to calculate the equilibrium value of the adsorbed dye per unit amount of adsorbent (q_e_ mg g^−1^). In addition, Equation (2) was used to compute the IV2R removal percentage (%).

(1) $$q_{e}=\frac{\left(C_{i}-C_{f}\right)\times V}{W}$$

(2) $$\mathrm{Percentage}\;\mathrm{removal}\;\left(\%\right)=\frac{\left(C_{i}-C_{f}\right)}{C_{i}}\times 100$$

where the concentrations of IV2R (mg g^−1^) at the initial ions of adsorption and equilibrium, respectively, are C_i_ and C_f_; m is the adsorbent mass (g); and V is the volume of the solution (L).

#### 2.5.2. Adsorption Kinetics, Equilibrium, and Thermodynamic Studies

The adsorption kinetics analysis showed the adsorption rate, the performance of the adsorbent, and the mass transfer mechanisms. Adsorption dynamics must be understood to design effective adsorption systems. 

Pseudo-first-order, pseudo-second-order, intraparticle diffusional, and Elovich models were used to evaluate the adequacy of different models for predicting adsorption kinetics. The IV2R adsorption kinetics of ZnO-NP samples were investigated at initial concentrations of 10 mg L^−1^, and 0.02 g dry adsorbent was mixed with 50 mL IV2R solution in batch studies carried out in a shaker for 200 rpm at 25 °C for 15, 30, 60, 120, and 180 min.

Each adsorption system (adsorbent material and adsorbate) had its isotherm, and the amount of the adsorbed adsorbate on an adsorbent was determined by ZnO-NPs and the characteristics of the solution. The most applicable models used to fit the kinetic sorption experiments were Lagergren’s pseudo-first-order model (Equation (3)) [43], a pseudo-second-order model (Equation (4)) [44], intraparticle diffusion (Equation (5)) [45], and the Elovich model (Equation (6)) [46]:

Log (q_e_ − q_t_) = log q_e_ − k_1_t/2.303 (3)

t/q_t_ = 1/K_2_ q_e_^2^ + t/q_e_ (4)

q_t_ = K_dif_ t^1^^/2^ + C (5)

(6) $$q_{t}=\frac{1}{\beta }\;\ln\;(\alpha \beta )\;+\;\ln(t)\frac{1}{\beta }$$

where q_e_ (mg g^−1^) is the quantity of dye adsorbed at equilibrium, and q_t_ (mg g^−1^) is the amount of dye adsorbed at a time (t). The pseudo-first-order and pseudo-second-order adsorption rate constants are k_1_ (min^−1^) and k_2_ (g mg^−1^ min), respectively. The intraparticle diffusion rate constant is determined using the slope of the regression line, and K_dif_ (mg g^−1^ min^0.5^) is the intercept. The Elovich constants are α (mg g^−1^ min^−1^) for the initial sorption rate and β (g mg^−1^) for the surface coverage and chemisorption activation energy. Nonetheless, the isotherm studies were performed using 0.02 g of dry adsorbent at initial IV2R concentrations of 10, 20, 40, 60, and 80 mg L^−1^, with enough time for adsorption equilibrium (180 min). Furthermore, thermodynamic studies of the adsorption experiments were carried out using the same approach at 25, 30, 35, 45, and 55 °C.

#### 2.5.3. Analysis of Errors

Traditional linear regression approaches for determining isotherm parameters appeared to provide a satisfactory fit for the data of the experiments. Different error functions of nonlinear regression were employed to calculate the constant model parameters, and they were compared to those determined from less accurate, linearized data-fitting techniques to evaluate the fit of the isotherm equations to the experimental data. The chi-square test and the residual root mean square error (RMSE) were utilized. *RMSE* is a term that can be defined as:

(7) $$\mathrm{RMS}=100\sqrt{\frac{1}{N}}\times \sqrt{\overset{N}{\underset{I=1}{\sum }}\left(1-\frac{q_{e},\mathrm{calc}}{q_{e},\mathrm{isotherm}}\right)2}$$

The subscripts “exp” and “cal” denote the experimental and calculated values, respectively, while n denotes the number of experimental isotherm observations and *N* is the number of the experimental data. 

Moreover, X^2^ is a small number of the data from the model calculation that is closest to the experimental data; the X^2^ value can be used to gauge how well the model fits. The better the curve fitting, the lower the RMSE value. The formula for the chi-square test (8) is as follows:

X^2^ = [(q_e, isotherm_ − q_e, calc_)^2^]/q_e, isotherm_ (8)

## 3. Results and Discussion

### 3.1. ZnO-NP Characterization

#### 3.1.1. FTIR

**Figure 1:**
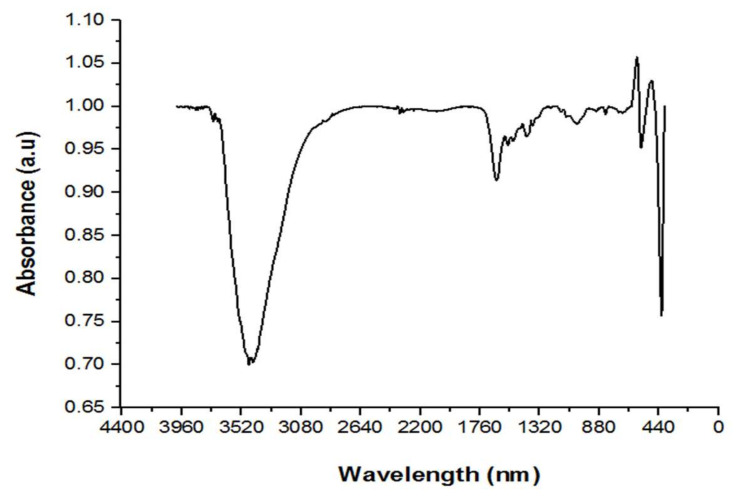
FTIR spectra of green ZnO-NPs.

The FTIR spectra of synthesized zinc oxide nanoparticles in the range of 400–4000 cm^−1^ are shown in Figure 1. A peak at about 3465 cm^−1^ that was assigned to N–H overlapped with a stretching vibration of hydroxyl groups (–OH) in the sample spectra. The weak absorption bands seen between 2344 and 2365 cm^−1^ corresponded to C–O stretching, which is one of the carboxylic group’s distinctive peaks [47]. The bands detected at 1638 cm^−1^ could be attributed to the samples’ C=O. The C=C stretching of aromatics was represented by the intensity peaks at 1553 and 1517 cm^−1^. The C–C stretching vibration of aromatic ring compounds caused the bands to be around 1416 cm^−1^. C–H asymmetric vibration was related to the bands at 1373 cm^−1^. C–O stretching vibration was assigned to the peaks of 1124 and 1038 cm^−1^ in the synthesis spectra. A band at 419 cm^−1^ confirmed the production of ZnO-NPs [48]. C–H bending vibration was associated with peaks at 832, 708, and 734 cm^−1^. Finally, the peak in the 400–600 cm^−1^ range was due to stretching vibrations of Zn–O [45,49]. The metallic group was responsible for the peak at 571 cm^−1^ in the FTIR diagram of the synthesized adsorbent. The presence of ZnO in the adsorbent was responsible for this peak [50]. In fact, electrons from either the C=O or C=C groups could transfer to the Zn^2+^ ions’ free orbital. Some functional groups in this sample, such as N–H, O–H, C=C, and C–O, were favorable to the adsorption process [48].

#### 3.1.2. SEM

**Figure 2:**
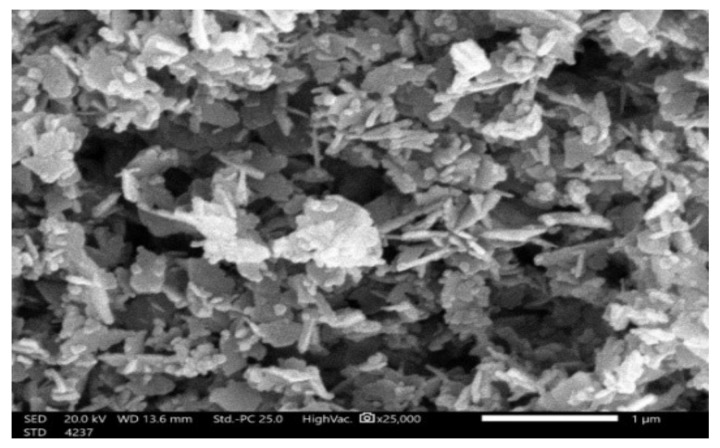
SEM images of synthesized adsorbent.

Scanning electron microscopy (SEM) is a vital technique for examining the textural structure, surface morphology, and particle size of an adsorbent. Figure 2 shows SEM pictures of the synthesized adsorbent at a magnification of ×25,000. The photographs demonstrate the likeness of microparticles with an average particle size of 1 µm and show that the outer surface of the ZnO-NP powder was composed of rod-like particles and small flower forms that were interconnected, producing holes and, therefore, yielding a large surface area. In addition, the microparticles on the sample surface had an uneven and porous texture, so there was a chance that IV2R molecules would be trapped. Additionally, the presence of these small particles increased the porosity and surface area of the synthetic adsorbent. This was supported by the measured surface area obtained for the synthetic adsorbent (113.751 m^2^g^−1^).

#### 3.1.3. BET

**Figure 3:**
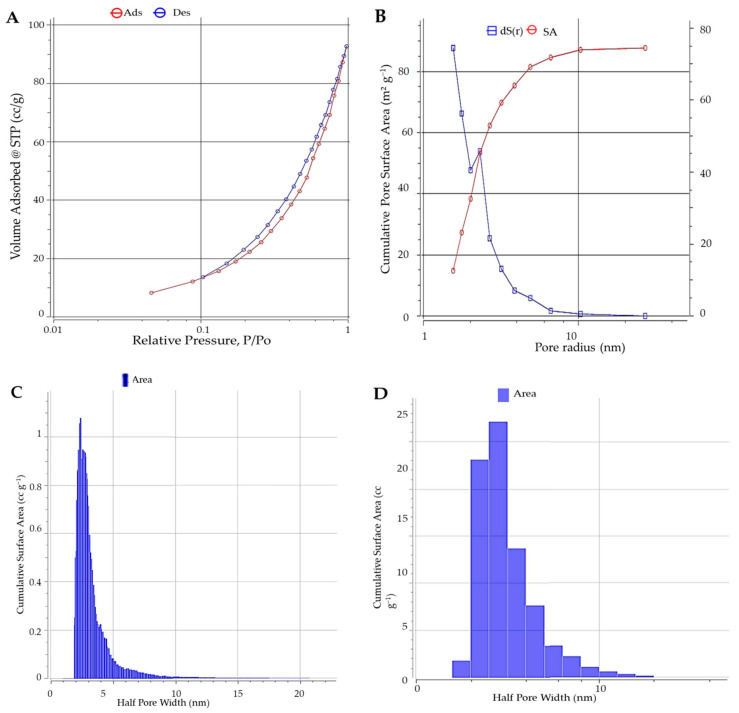
BET analyses and N_2_ adsorption–desorption isotherms of green ZnO-NPs: (**A**) isotherm–isotherm, (**B**) BJH adsorption results, (**C**) DFT method histogram of surface area (linear), and (**D**) DFT method histogram of surface area (log).

For BET, when the ratio is close to 1, the curve may reach a limiting value or climb if larger pores are present in micropore materials, which absorb more N_2_ at low relative pressures. The produced adsorbent’s N_2_ adsorption–desorption isotherm could be classified as a type I isotherm, as shown in Figure 3. The adsorption isotherm directly touched the line of P/Po = 1 in this diagram. The volumes adsorbed at a low relative pressure (P/Po 0.1), indicating that nitrogen molecules were mainly adsorbed on the micropores. The synthesized adsorbent had a BET surface area of 113.751 m^2^ g^−1^, whereas the BJH desorption was 82.23 m^2^ g^−1^. It had a large surface area, which gave this low-cost material a valuable property. Furthermore, the overall pore volume was 0.143 C^2^ g^−1^, and the average pore size was 2.527 nm, as presented in Table 2. The adsorbent had a high volume of broad micropores and a high proportion of micropore volume.

### 3.2. XRD Study 

**Figure 4:**
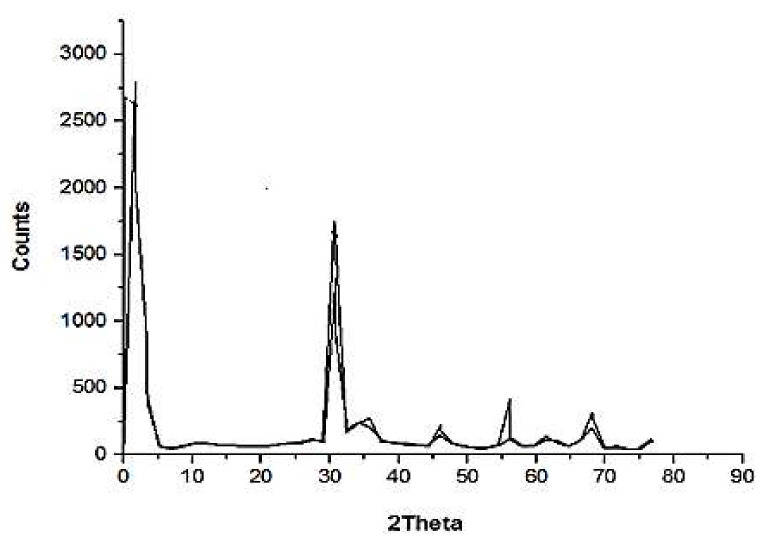
Pattern of XRD of synthesized ZnO.

The crystal structure of the produced material was investigated using X-ray diffraction [42]. ZnO-NPs’ X-ray diffraction pattern was discovered utilizing an XRD system with a range of 0 to 100° A. The XRD patterns of green synthetic ZnO-NPs are displayed in Figure 4. The ZnO powder was extremely crystalline, according to the XRD peak, and all of the peaks were arranged in a way that corresponded to either the cubic shape of ZnO, which is the reference pattern for synthetic ZnO-NPs (65-2800), or the hexagonal structure of ZnO-NPs (05-20664), which is the reference pattern for green ZnO-NPs [41]. Additionally, the sharp and intense peaks showed the crystal structure of the produced ZnO nanoparticles [51]. Additionally, a mixed hexagonal and cubic phase was visible in the results when compared to the previous reference patterns.

### 3.3. UV

**Figure 5:**
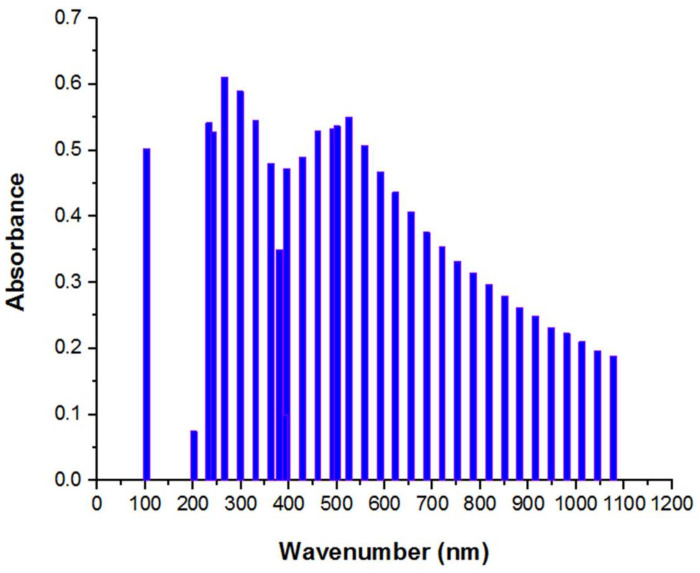
UV-vis spectra of green ZnO-NPs.

The key benefits of this method are that it is readily available, affordable, and simple to use. Since the sample was nontoxic, thus there was no pollution. The wavelength range of the metal nanoparticles was determined with UV-vis, as shown in Figure 5. The maximal absorbance was shown by the highest peak. The production of zinc oxide nanoparticles was confirmed by the UV spectrometer absorption peaks in the 300–400 nm range. 

These peaks likewise reflected the particles’ narrow particle size distribution and nanoscale size. The absorption peak at 243 nm, which is associated with π→π* transitions in the sesquiterpene system, could hardly be seen in the UV spectra of the ZnO-NPs, which could be due to a change in the sesquiterpene structure and absence of this π→π* transition [52].

### 3.4. Influence of Experimental Parameters on the Adsorption Mechanism

#### 3.4.1. pH

**Figure 6:**
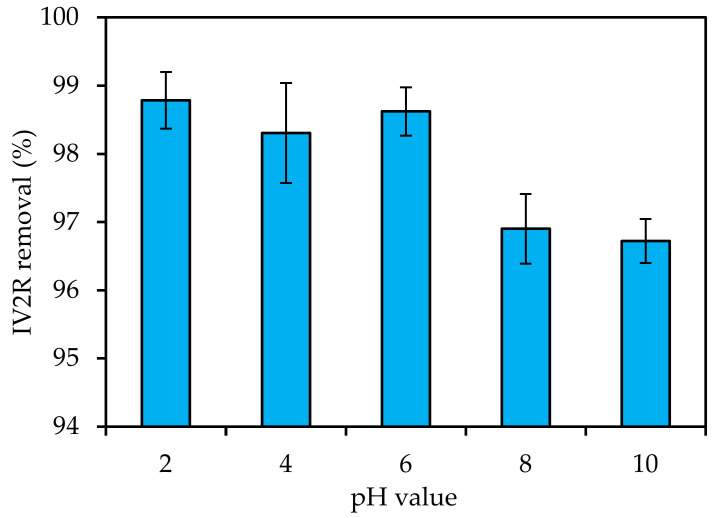
Influence of pH on removal of IV2R using green ZnO-NPs (adsorbent amount: 0.02 g; temperature: 25 ± 2 °C; initial volume: 50 mL; adsorbent dosage: 0.02 g; initial dye concentration: 10 mg L^−1^; and contact time: 180 min).

The pH of the solution had an impact on the surface charge of the adsorbent, the levels of ionization of various pollutants, the dissociation of functional groups on the active sites of the adsorbent, and the structure of the dye molecule [53]. As a result, during the dye adsorption process, the pH of the solution was a key parameter. The IV2R molecules’ ability to ionize and the surface properties of the adsorbent were both influenced by pH, hence the effectiveness of the adsorption was tested at varied solution pH levels. The effect of the initial pH of the solution on IV2R adsorption is shown in Figure 6. IV2R adsorption increased from pH 2 to 6 and then decreased. It was found from the results that the IV2R dye elimination was maximal at 98.78% for pH 2. This could be because, at low pH values, the external surface of the adsorbent material was positively charged via absorbing H^+^ ions [54]. Furthermore, the change in surface charge of the adsorbent could cause a high percentage of removal at pH 2 and 6. As the pH of the IV2R solution increased, the adsorbent’s surface became negatively charged, aiding the electrostatic-attraction-based adsorption of the positively charged cationic dye [27]. 

#### 3.4.2. ZnO-NP Dosages

**Figure 7:**
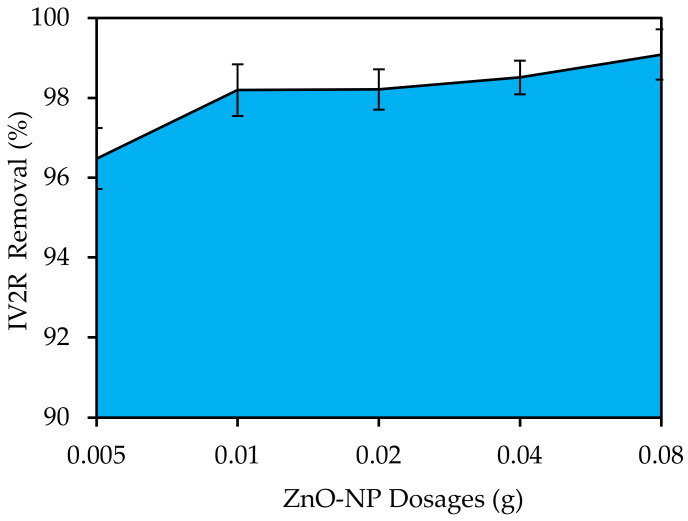
Influence of ZnO-NP adsorbent dosage on removal of IV2R (pH: 6; temperature: 25 ± 2 °C; initial volume: 50 mL; initial dye concentration: 10 mg L^−1^; and contact time: 180 min).

The dose of the adsorbent determined its capacity for a specific initial concentration of the adsorbate under the operating conditions. Figure 7 depicts the effect of adsorbent dosage on IV2R removal. For this study, adsorbent dosages ranging from 0.005 to 0.8 g were added to 50 mL of IV2R solution. Because of the increase in the number of adsorption sites, the percentage of the removal of IV2R increased as the adsorbent dosage increased. As expected, the percentage of elimination increased from 96.47% to 99% when the adsorbent dosage increased from 0.005 to 0.8 g. As a result, only 0.8 g of adsorbent were required to remove 99% of the IV2R dye when the quantity of the adsorbent material was augmented. It is evident that, as the adsorbent dosage was raised, the IV2R dye ions’ capacity for adsorption grew quickly due to the increased availability of exchangeable sites and surface area, and that, after equilibrium was established, increasing these sites had little effect [55], as the adsorption process is a surface phenomenon. According to the results, as the adsorbent dosage was increased, the removal efficiency increased, but the adsorption level capacity decreased. This was caused by the polysaccharide and fiber-like elements found in the algae cell walls, the adsorbent active surface, and dynamic elements such as an increased level of contact and an increased number of free bonds on the adsorbent surface, which could be specified using adsorption isotherms and synthetic equations, according to Aziz et al. [52].

#### 3.4.3. Contact Times

**Figure 8:**
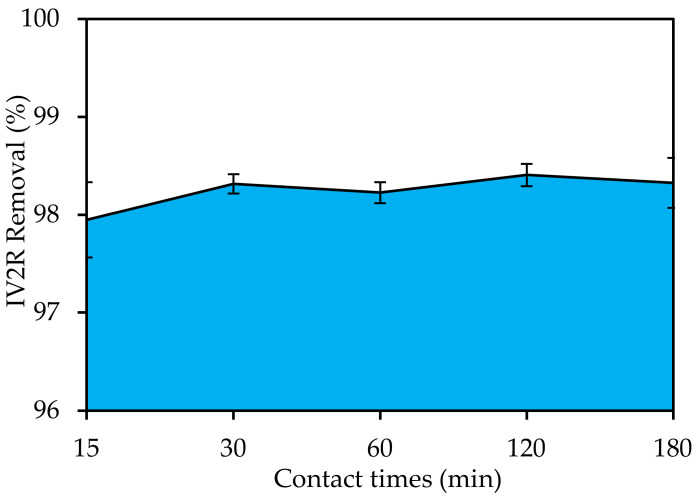
Influence of contact time on IV2R removal by green ZnO-NPs (pH: 6; temperature: 25 ± 2 °C; initial volume: 50 mL; initial dye concentration: 10 mg L^−1^; and adsorbent dosage: 0.02 g).

The effect of contact time on IV2R adsorption in solutions with 10 mg L^−1^ initial concentrations is shown in Figure 8. It reveals that the adsorption rate rose significantly with increasing contact time to reach 98.4% after 120 min, and then the IV2R dye reached adsorption equilibrium. Furthermore, once equilibrium was attained, the adsorption capacity remained constant. This was most likely caused by the IV2R ion saturation of nano-adsorbent surfaces, which was followed by adsorption and desorption processes [52]. Furthermore, it occurred due to the adsorbent’s huge surface area, as well as strong attraction forces between the adsorbent and dye molecules. The rate of removal increased at first and then lowered to a near-equilibrium condition after 30–120 min. According to Abate et al. [56], the maximum number of active sites is initially available but becomes saturated over time. The remaining vacant surface sites are challenging to occupy as a result of the formation of a repulsive force between the adsorbate on the solid surface of the adsorbent and the adsorbate on the bulk phase (solutions), as well as the saturation of the active site of the adsorbent.

#### 3.4.4. Initial IV2R Concentration

**Figure 9:**
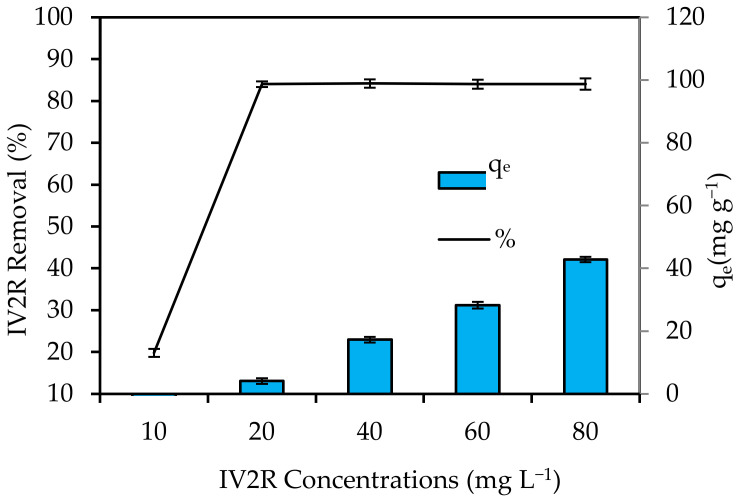
Influence of initial dye concentrations on removal of IV2R by green ZnO-NPs (pH: 6; temperature: 25 ± 2 °C; initial volume: 50 mL; adsorbent dosage: 0.05 g; and contact time: 180 min).

Figure 9 shows the effects of initial dye concentrations ranging from 10 to 80 mg L^−1^. With increasing initial concentrations, the IV2R adsorption increased progressively because the initial dye concentration was crucial to the adsorption process, as it served as a driving force for transferring dyes from the aqueous solution to the solid surface. The ratio of IV2R molecules to the accessible surface area increased as concentrations rose. As a result, the adsorption process was influenced by the dye concentration at the start. The available active sites of the adsorbent were degraded at high dye concentrations, and the amount of adsorbed IV2R decreased. However, when the initial concentration of IV2R dyes increased, the adsorption capacity (q_e_) significantly increased. The adsorption capacity (q_e_) increased from 1 to 42.1 mg g^−1^ as the dye concentration was increased from 10 to 80 mg L^−1^. Therefore, the interaction between the dye molecules and the surface of the adsorbent was increased with increasing initial IV2R dye concentrations [57]. 

#### 3.4.5. Temperature

**Figure 10:**
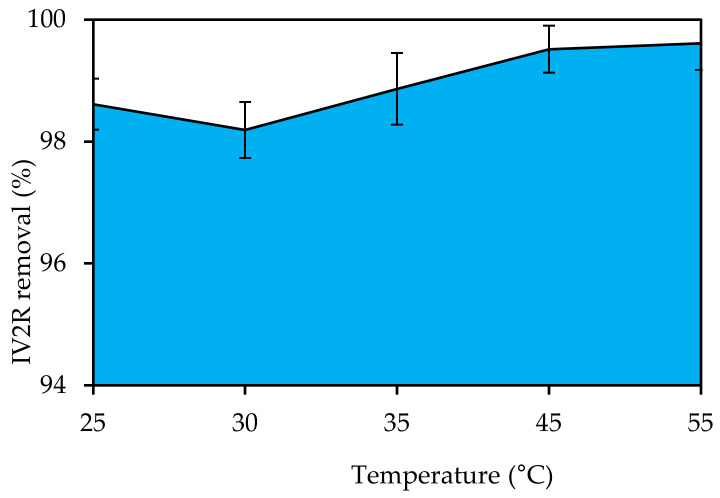
Influence of temperature on IV2R removal by green ZnO-NPs (pH: 6; initial volume: 50 mL; initial dye concentration: 10 mg L^−1^; adsorbent dosage: 0.02g; and contact time: 180 min).

The influence of temperature on IV2R removal by the synthesized adsorbent was investigated in the range of 25–55 °C, as shown in Figure 10. The IV2R removal increased as the temperature rose from 30 to 55 °C, and it was nearly constant at higher temperatures. At 55 °C, the highest adsorption of IV2R was observed at higher temperatures, and the removal was practically constant, with a percentage of clearance of 99.61%. The fact that adsorption increased with the temperature increase from 30 to 55 °C implied that IV2R adsorption is an endothermic process. Changing the dye adsorption by altering the temperature revealed that, as the temperature rose, the adsorbed surface activity rose and that IV2R adsorption is mostly controlled by chemical forces [58]. 

### 3.5. Adsorption Isotherms 

**Figure 11:**
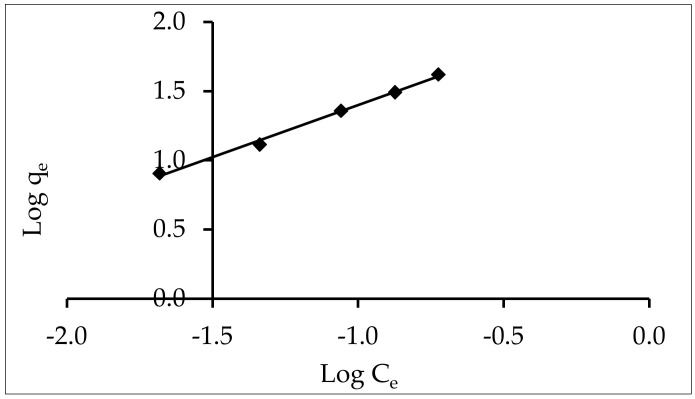
Freundlich equation for the sorption of IV2R onto Zn-ONPs.

**Figure 12:**
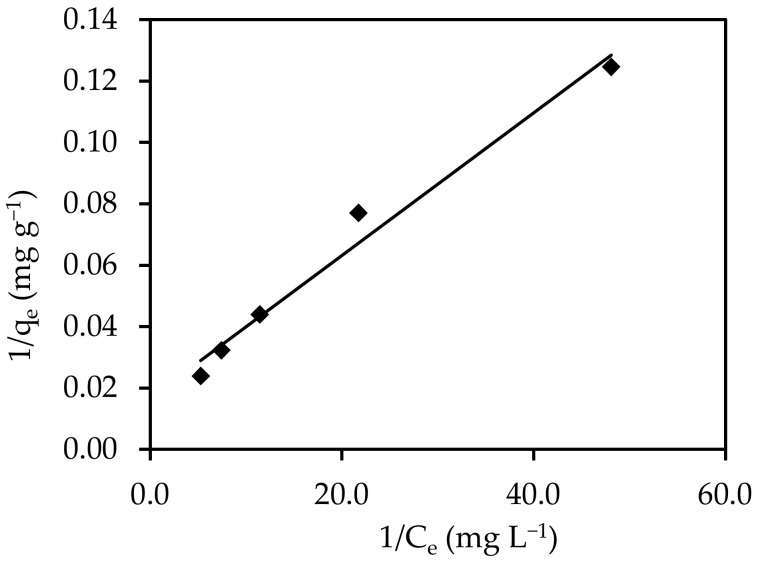
Langmuir model for the adsorption of IV2R onto ZnO-NPs.

**Figure 13:**
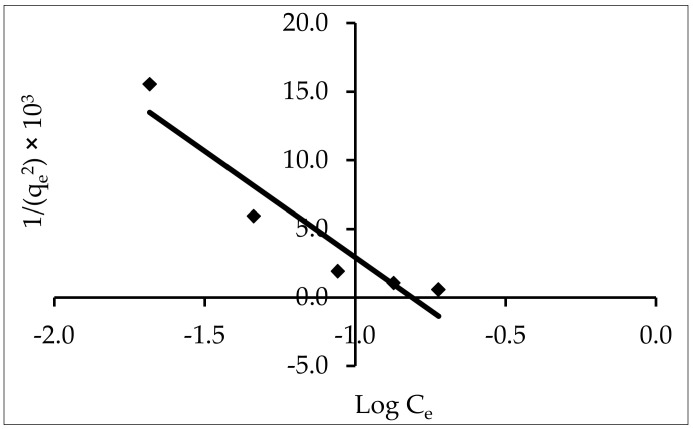
Harkins–Jura adsorption isotherm for the adsorption of IV2R onto ZnO-NPs.

**Figure 14:**
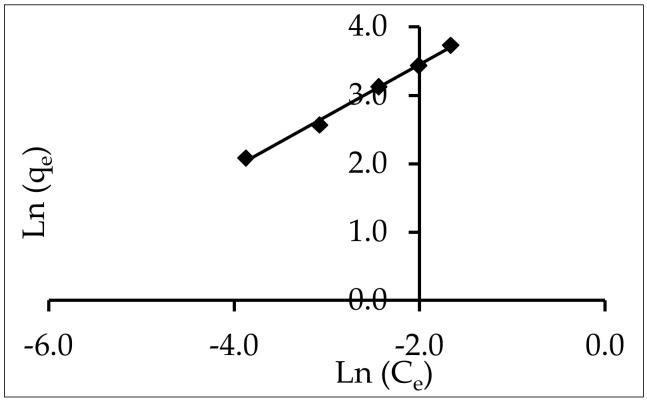
Halsay adsorption isotherm for the adsorption of IV2R onto ZnO-NPs.

**Figure 15:**
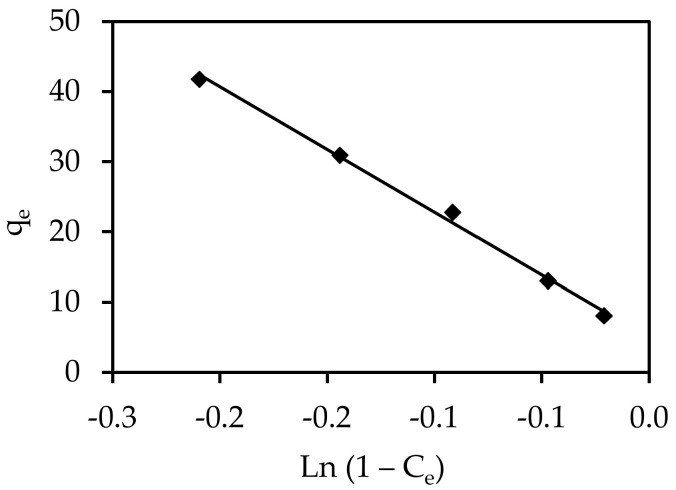
Smith isotherms for the adsorption of IV2R onto ZnO-NPs.

**Figure 16:**
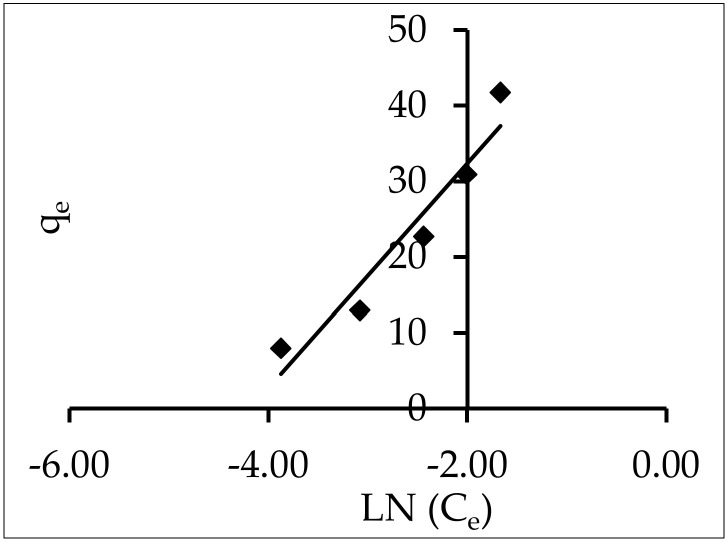
Tempkin isotherms for the adsorption of IV2R onto ZnO-NPs.

Adsorption isotherms were used to determine the interaction between the adsorbent and the dye, as well as the dye’s adsorption capacity. The first empirical technique used to investigate the nature of adsorption was the isotherm shape. The IV2R adsorption isotherms of ZnO-NPs are shown in Figure 11, Figure 12, Figure 13, Figure 14, Figure 15 and Figure 16. The fitting of isotherm data to various isotherm models aided in the development of an appropriate model for the adsorption process, as well as the estimation of the adsorbent’s adsorption capacity. The equilibrium results were compared with ZnO-NPs, which have a large surface area, to better understand the synthesized adsorbent adsorption capacity. Langmuir, Freundlich, Tempkin, Harkins–Jura, Halsey, and Sips isotherms were used to assess the experimental equilibrium data. The four equilibrium isotherm models utilized for IV2R adsorption on the generated ZnO-NPs presented in Table 3 yielded all the correlation coefficients, R^2^ values, and adsorption parameters at equilibrium.

#### 3.5.1. Freundlich Isotherm

Adsorption varies in a manner that is directly proportional to pressure, according to the Freundlich isotherm. This empirical equation, which assumes that different locations have various adsorption energies involved, describes the multilayer adsorption of heterogeneous systems [44]. The isotherm’s linear model can be written logarithmically, as demonstrated in the following equation:

Log q_e_ = Log K_f_ + 1/n Log C_e_ (9)

The intercept and slope of the plot of log C_e_ against log q_e_ (Figure 11) can be used to determine the values of parameters K_f_ and n (listed in Table 3), where K_f_ (1 g^−1^) is the Freundlich adsorption capacity constant, and 1/n is the adsorption intensity constant that varies with the heterogeneity of the adsorbate. The Freundlich isotherm, with R^2^ = 0.995, was determined to be the best fit for the adsorption experimental data; the high correlation coefficient suggested a high affinity between the ZnO-NP adsorbent surface and the IV2R dye, which played a large role in the adsorption mechanism. This could be due to the nonhomogeneous nature of the adsorbent surface, which could include multiple types of sorption sites [37]. The adsorption favorability is determined by the magnitude of the (n) parameter. A favorable adsorption condition is defined as n > 1 in the range of 1–10 [38], (in the present study, it was 1.32 for IV2R dye). Moreover, Table 3 shows that the Freundlich isotherm fit the experimental data well, as evidenced by the RMSE and chi-square values. This result also indicates that IV2R adsorption on ZnO nanoparticles was based on multilayer adsorption.

#### 3.5.2. Langmuir

The pressure dependence of molecule (adsorbate) adsorption is demonstrated by the Langmuir adsorption isotherm model. When the energy of adsorption is constant, a saturated monolayer of adsorbate molecules is present on the adsorbent surface, there is no migration or contact between the adsorbate molecules on the surface plane, and maximum adsorption occurs [59]. The Langmuir isotherm model’s linear expression is defined using the following Equation (10).

1/q_e_ = 1/(Ka q_m_ C_e_) + 1/q_m_ (10)

The values of Ka and qm can be determined from the slope and intercept of the linear graph of 1/C_e_ against 1/q_e_ (Figure 12), where qm (mg g^−1^) is the maximum adsorption capacity of dye per unit mass of sorbent to form a complete monolayer on the surface-bound adsorption, and Ka (L mg^−1^) is the Langmuir energy constant, which is related to the heat of adsorption. The Langmuir constants are listed in Table 3 (q_m_ = 59.88 mg g^−1^ and Ka = 7.26 L mg^−1^). The Langmuir isotherm’s essential properties can be stated in terms of a dimensionless constant separation factor R_L_, which is given in Equation (11):

(11) $$R_{L}=\frac{1}{1+bC_{i}}$$

where C_i_ (mg L^−1^) denotes the greatest initial adsorbate concentration. 

The value of R_L_ shows the type of isotherm to be either linear (R_L_ = 1), favorable (0 < R_L_ < 1), unfavorable (R_L_ > 1), or irreversible (R_L_ = 0). In this study, the value of R_L_ was determined to be 0.038, and a value between 0 and 1 denoted favorable adsorption, which indicated that IV2R adsorption on the surface of ZnO-NPs was advantageous. Because the Langmuir equation assumes that the adsorbent surface is energetically homogeneous with high root mean square error (RMSE) and chi-square (X^2^) values, the fact that the Langmuir isotherm fit the experimental data well could be due to the uniform dispersion of active sites on the ZnO-NPs.

#### 3.5.3. Harkins–Jura 

Multilayer adsorption is explained by the Harkins–Jura adsorption isotherm, which is explained by the presence of heterogeneous pore distribution. The Harkins–Jura adsorption isotherm can be expressed as:

(12) $$\frac{1}{{\mathrm{qe}}_{}^{2}}=\left(\;\frac{B_{2}}{A}\right)-\left(\frac{1}{A}\right)\log C_{e}$$

where the isotherm constants are A and B.

The value of 1/q_e_ is plotted against log C_e_; the isotherm constants are B (intercept/slope; mg^2^ L^−1^) and A (1/slope; g^2^ L^−1^) (Figure 13).

For ZnO-NPs, this model exhibited the best fit for the results, with R^2^ equal to (0.900). The obtained results showed that multilayers of adsorption formed and that the ZnO-NP adsorbent was porous. In addition to considering the existence of a heterogeneous pore distribution, the Harkins–Jura isotherm is related to the Freundlich model. Because of large RMSE and X^2^ values, the Harkins–Jura model was less suitable to describe IV2R adsorption onto ZnO-NPs.

#### 3.5.4. Halsay

The experimental data that fit this equation attest to the adsorbent’s heterosporous nature, and the equation is appropriate for multilayer adsorption. The slope and intercept of a plot of ln q_e_ vs. ln C_e_, as illustrated in Figure 14, can be used to determine n and K with the following equation:

(13) $$\mathrm{Ln}\left[-\mathrm{Ln}\left(1-C_{e}\right)\right]=\mathrm{LnK}+\left(\;\frac{1}{n}\;\right){\mathrm{Lnq}}_{e}$$

Table 3 lists the model parameters. Because the Halsay model assumes a multilayer behavior for the adsorption of an adsorbate onto an adsorbent, it may also be used to represent the adsorption of IV2R onto ZnO-NPs. This was supported by high RMSE and X^2^ values (Table 4).

#### 3.5.5. Smith 

The Smith model is suitable for multilayer adsorption and heteroporous solids. This model is frequently derived using the equation shown below:

q_e_ = W_bS_ − W_S_ Ln (1 − C_e_) (14)

where W_bS_ and W_s_ are the Smith model parameters.

The Smith model is applicable for heteroporous solids and multilayer adsorption, as well as explaining the adsorption isotherms of biological substances such as cellulose and starch. A plot of q_e_ vs. Ln (1− C_e_) can be used to solve the Smith model, as shown in Figure 15. The sorption isotherms were properly represented by the Smith model across the entire range of water activity. The Smith equation, on the other hand, was adsorption. The correlation coefficients and isotherm constants varied from R^2^ = 0.995.

#### 3.5.6. Tempkin 

Lastly, the Tempkin isotherm considers the adsorption process’s indirect adsorbent–adsorbate interactions [60]. Due to adsorbent–adsorbate interactions, the heat of adsorption of all the molecules in a layer drops linearly with coverage. It can be written as q in the linear form [61]:

q_e_ = B Ln A + B Ln C_e_ (15)

where B = (RT)/b is the universal gas constant of 8.314 J (mol K^−1^), and T is the absolute temperature in Kelvin. The heat of adsorption is proportional to the constant b. The isotherm constants A and B can be calculated from the slope and intercept of a plot of q_e_ vs. ln C_e_ (Figure 16).

The computed A was 14.65 L g^−1^, which was the equilibrium binding constant corresponding to the maximal binding energy, as shown in Figure 14. The heat of adsorption for the ZnO nanoparticles was related to constant B, which was equal to 65.36 J mol^−1^. Moreover, the Tempkin model was shown to be unsuitable for IV2R adsorption onto ZnO-NPs by comparing the values of the error functions.

### 3.6. Models of Sorption Kinetics

Adsorption is a physiochemical process in which a solute (adsorbate) is transferred from the liquid phase to the adsorbent surface through mass transfer. The study of adsorption kinetics is desirable because it gives information about the adsorption mechanism, which is critical for process efficiency. 

#### 3.6.1. Pseudo-First-Order Model

**Figure 17:**
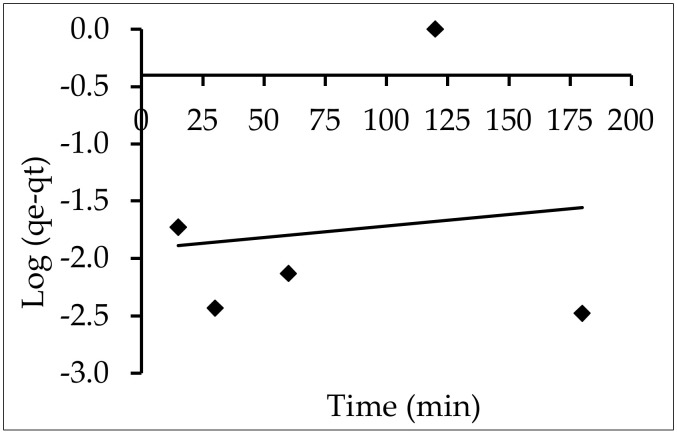
The pseudo-first-order equation for the elimination of IV2R onto ZnO-NPs.

The first model of the adsorption rate based on adsorption capacity was the pseudo-first-order kinetic model. Log (q_e_ − q_t_) against (t) plots showed a linear relationship between k_1_ and q_e_, as measured by the slope and intercept, respectively. R^2^ = 0.018 indicated that the correlation coefficients were not good enough. Furthermore, the calculated value of q_e_ from the equation, 82.41 mg g^−1^, differed with the experimental value of 3.99 mg g^−1^) Table 4 and Figure 17). 

#### 3.6.2. Pseudo-Second-Order Model

**Figure 18:**
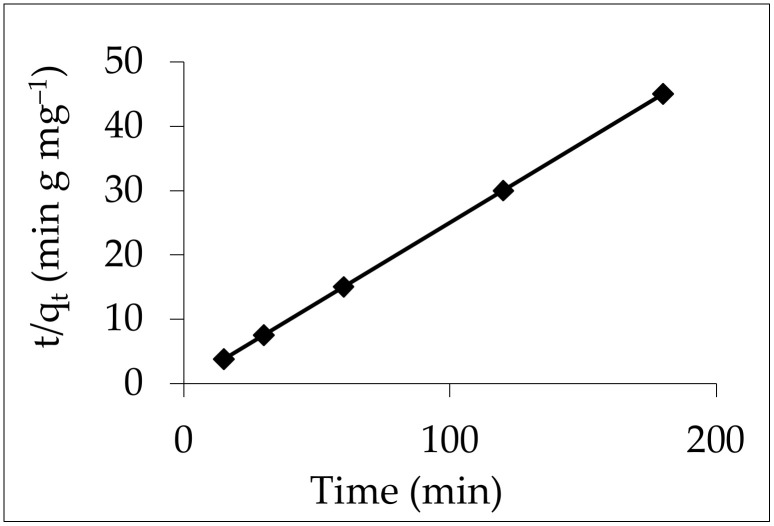
The pseudo-second-order equation for the elimination of IV2R onto ZnO-NPs.

A pseudo-second-order model was used to further examine the experimental kinetic data. By graphing t/q_t_ against t for IV2R, a straight line was obtained. The slope and intercept of the curve in Figure 18 were used to calculate the second-order rate constant and q_e_ values, which are reported in Table 4. The correlation coefficients (R^2^) for IV2R adsorption onto ZnO-NPs were determined to be equal to one. The kinetics of IV2R adsorption onto ZnO-NPs could be well-represented by the second-order equation. This showed that chemisorption, which involves valent forces and the sharing or exchange of electrons between the adsorbent and the adsorbate, could be the rate-limiting phase in this sorption process [44].

#### 3.6.3. Model of Interparticle Diffusion

**Figure 19:**
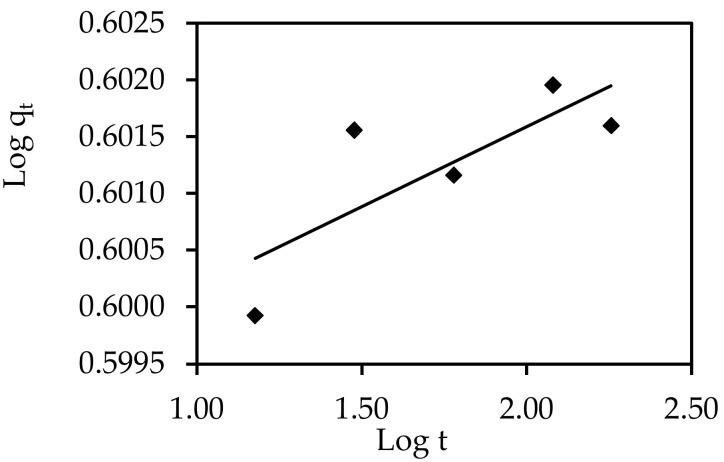
Interparticle diffusion equation for the elimination of IV2R onto ZnO-NPs.

The overall adsorption rate in a porous adsorbent, according to the literature, must account for the three processes of external mass transfer, intraparticle diffusion, and adsorption on active sites inside the pores. Film or intraparticle diffusion, or a mixture of both mechanisms, affects the overall rate of adsorption. Plotting (q_t_) versus (T^0.5^) yields a linear relationship, and the slope and intercept can be used to calculate the K_dif_ parameter [62]. Table 4 lists the values of K_dif_ and the R^2^ correlation coefficients determined from the intraparticle diffusion plots. The fact that the linear curve in Figure 19 did not pass through the origin suggests that boundary layer diffusion had some influence on adsorption and that intraparticle diffusion was not the primary factor dictating the rate [63]. The discrepancy in mass transfer rates between the starting and final stages of adsorption could be the cause of this deviation [64].

#### 3.6.4. Elovich Model 

**Figure 20:**
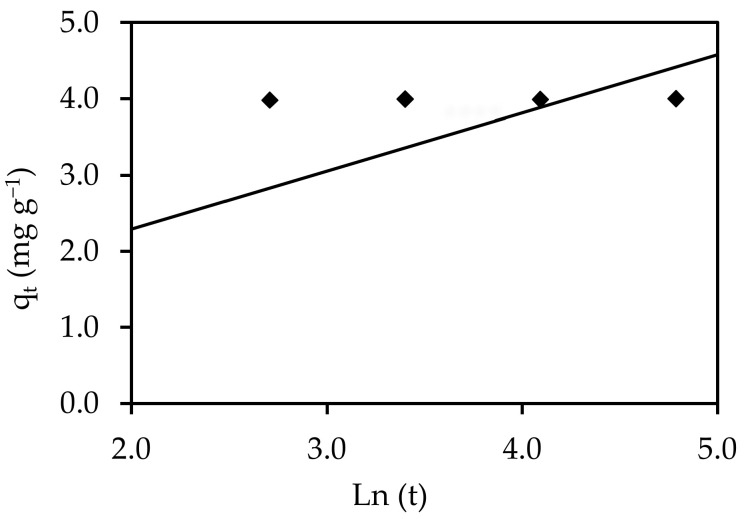
The Elovich equation for the elimination of IV2R onto ZnO-NPs.

One of the most effective models for explaining the kinetics of gas chemisorption onto solid systems is the simple Elovich model. However, it has recently been used to describe the process of contaminants adsorbing from aqueous solutions. The plot of q_t_ against Ln (time) for IV2R sorption onto ZnO-NPs is shown in Figure 20. The estimated Elovich equation parameters were derived from the slope and intercept of the linearization of the simple Elovich equation. The number of adsorption sites available was indicated by the value of β. Table 4 shows the adsorption quantity when Ln t was equal to zero, i.e., the adsorption quantity when t equaled 1 h. This value added knowledge of the first step’s adsorption behavior. In addition, it is obvious from this diagram that the Elovich equation did not fit the experimental data well. For the Elovich model, a small β (mg g^−1^ min^−1^) (desorption constant) value (<1) indicates an irreversible adsorption method [65]. Nevertheless, the average β parameter of 1.31 (g mg^−1^) achieved in this study indicated that the adsorption process was readily reversible.

### 3.7. Thermodynamic Studies

The influence of temperature on adsorption capacity was investigated using thermodynamic analyses. The following Equations (16)–(19) were used to determine the Gibb’s free energy change (ΔG°), the enthalpy change (ΔH°), and the change in entropy (ΔS°):

Kd = qe/Ce(16)

ΔG° = −RT Ln K_d_ (17)

ΔG° = ΔH° − T ΔS°, or(18)

**Figure 21:**
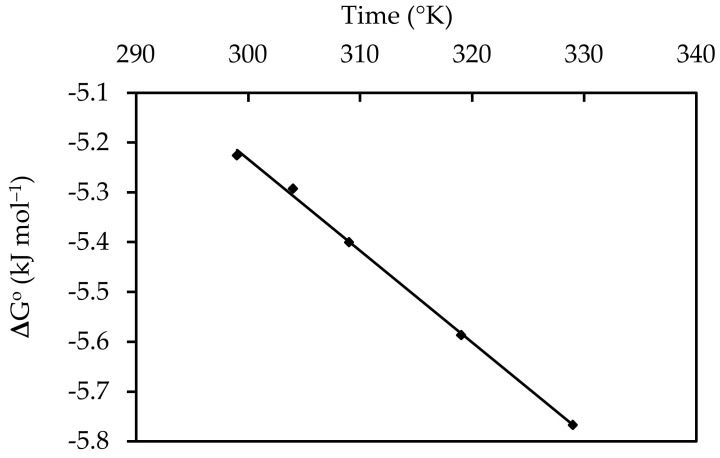
Thermodynamic behaviors of the removal of IV2R onto ZnO-NPs.

ΔG° = T (ΔS°) + ΔH°(19)

where q_e_ is the solid phase equilibrium concentration in milligrams per liter (mg L^−1^), C_e_ is the equilibrium concentration in milliliters per liter (mg L^−1^), T is the temperature in degrees Celsius, and R is the gas constant (8.314 J mol^−1^ K). In addition to ΔG°, which may be obtained from Equation (17) as shown in Figure 21, the values of ΔH° and ΔS° were determined using the intercept and slope of the displayed curve of T vs. ΔG° from Equations (18) or (19). Table 5 lists the thermodynamic parameters. The fact that the change in free energy (ΔG°) was negative showed that the adsorption process was feasible and that IV2R adsorption onto ZnO-NPs occurred spontaneously at the temperature range investigated. Furthermore, a positive enthalpy change (ΔH°) suggested that the adsorption was chemical and endothermic. The entropy value (ΔS°) was negative. This showed that, during the adsorption process, the unpredictability between the solid–solution contacts was reduced, as presented in Table 5.

### 3.8. Dye Removal Mechanism

The mechanism of adsorption is one of the most important tools to know when developing a system. The most prevalent adsorption mechanisms are chemical adsorption, physical adsorption, and ion exchange. The adsorption mechanism can be examined through the modelling of the data from the adsorption equilibrium and an analysis of the empirical results. It is feasible to indicate the basic mechanisms of adsorption for dye on an adsorbent by taking into consideration data such as pore size distribution, thermodynamics, and equilibrium results. Electrostatic interaction was the main force at work between the adsorbent and the adsorbed dye. Hydrogen bonding, hydrophobic interactions, and interactions of the aromatic rings of the ZnO-NPs with the aromatic rings of the dye all played significant roles in the adsorption process between the surfaces of the ZnO-NPs and IV2R. Furthermore, the aromatic rings of IV2R molecules interacted with the ZnO-NPs’ C=O, OH, NH, and phenyl groups, which acted as adsorption sites. The evaluation of the generated adsorbent extraction also revealed the presence of ZnO. In aqueous solutions, zinc oxide is virtually insoluble and acts as a weak base. In this experiment, the surface synthesis of ZnO nanoparticles was primarily Zn (OH)^+^ at pH = 6.0 As a result, ionic bonding between the negatively charged functional groups of IV2R dye (−SO_3_^−^) and the positively charged core of ZnO (Zn(OH)^+^) may be the fundamental mechanism of interaction between IV2R dye and ZnO. As a result, IV2R adsorption by the synthesized adsorbent could be a chemisorption process.

### 3.9. Synthetic Dye Effluent Treatment

According to the adsorption data (kinetic and equilibrium), the ZnO-NP material was very effective at removing IV2R from aqueous solutions, suggesting that they might be used to treat actual effluents. As a result, ZnO-NPs were put to the test for treating IV2R actual and synthetic dye effluents. UV-vis spectra of the effluents were used to calculate the ZnO-NP removal percentage of the dye mixture in the effluents. It was explored whether widely a available water treatment could be used as a low-cost material for removing color from real textile wastewater. In the batch test, dried ZnO-NPs were utilized as an adsorbent. The results showed that the actual effluents were removed with the highest amount of color at 95.6% when compared to synthetic IV2R dye with distilled water at almost 99.6%. Industrial effluents may contain toxic compounds and compounds that contain a variety of organic and inorganic materials that are possibly mutagenic or carcinogenic, as well as acids, alkalis, and radioactive elements.

### 3.10. Comparison with Other Adsorbents

Table 6 compares the maximal IV2R adsorption capabilities of the synthesized adsorbent, ZnO-NPs, with those of several other adsorbents, such as modified biomasses, activated carbons, and zeolites. The synthesized adsorbent had a greater maximum adsorption capacity (59.88 mg g^−1^) than the other adsorbents listed in Table 6. The Cu(II) ions in organic dye could be combined with the hydroxyl groups and amino groups of the adsorbent through a strong electrostatic attraction process. Consequently, the solid green ZnO-NPs could be used as favorable and cost-effective sorption material for eliminating IV2R ions from industrial wastewater.

## 4. Conclusions

The current study suggested that macro-algae’s ability to synthesize ZnO-NPs, which could be used to cure IV2R dye, makes them a strong contender. FTIR spectroscopy, SEM, UV-vis, and BET measurements were used to characterize the synthesis. The presence of ZnO-NPs was indicated by a 419 cm^−1^ FTIR band with a sharp, significant band at 571 cm^−1^. The synthetic adsorbent had a BET surface area of 113.751 m^2^ g^−1^. The starting dye concentration, pH, contact time, solution temperature, and adsorbent dosage were found to be the optimal process parameters for the saturation of accessible binding sites. The adsorption of IV2R on the synthetic adsorbent was aided by a temperature of 55 °C, a pH of 6, and a high adsorbent dosage. According to Langmuir, the adsorbent was a low-cost adsorbent with an IV2R adsorption capability of 59.88 mg g^−1^. Furthermore, ZnO-NPs had a 95% absorption rate for the treatment of the genuine effluents tested. For linearly and nonlinearly regressed data, the resulting isotherm data fit best with the Freundlich, Smith, Halsey, and Tempkin isotherm models, while the kinetic data fit best with a pseudo-first-order model. The computed thermodynamics parameters revealed that the adsorption system in concern was favorable, endothermic, and spontaneous. As a result, ZnO-NPs may be thought of as a possible adsorbent for removing IV2R dye from polluted water.

## Figures and Tables

**Table 1:** Physical characteristics of the dyes.

Characteristics	Value
Dye name	Ismate violet 2R
Wavelength (λ max)	550 nm
Mol. wt.	700
Molecular formula	C_22_H_14_N_4_O_11_S_3_CuCl
C.I. name	IV2R
Molecular structure	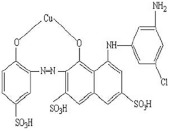

**Table 2:** BET surface area and porosity of ZnO-NPs.

Surface Characteristics	Value
BET surface area	113.751 m^2^ g^−1^
Langmuir method	215.295 m^2^ g^−1^
Average pore size	2.52797 nm
Total pore volume	0.14378 C^2^ g^−1^
DH desorption	84.0658 m^2^ g^−1^
BJH desorption	82.2329 m^2^ g^−1^
Average particle radius	1.1988 nm
Polarizability	1.46 (mL mol^−1^)
Surface atom density	13.1 (mol cm^2^)

**Table 3:** Correlation coefficients and isotherm model constants of adsorption of IV2R on ZnO-NPs.

RMSE	X^2^	Value	Isotherm Parameter	Isotherm Model
0.046	0.001	0.754	1/n	Freundlich
		34.30	K_F_ (mg^−1−1/n^ L^1/n^ g^−1^)	
		0.995	R^2^	
0.047	0.002	59.88	Q_max_ (mg g^−1^)	Langmuir
		7.26	K_a_	
		0.979	R^2^	
		0.038	R_L_	
14.995	65.439	0.06	A_HJ_	Harkins–Jura
		0.81	B_HJ_	
		0.894	R^2^	
0.054	0.001	0.753	1/n_H_	Halsey
		721	K_H_	
		0.995	R^2^	
0.004	0.000	4.940	W_bs_	Smith
		178.730	W_s_	
		0.995	R^2^	
16.192	76.305	14.65	A_T_	Tempkin
		65.36	B_T_	
		592.46	b_T_	
		0.926	R^2^	

**Table 4:** Adsorption kinetics of sorption of IV2R onto ZnO-NPs.

Model	Parameter	Value
First-order kinetic	q_e_ (calc.)	82.41
	K_1_ (min^−1^)	4.66 × 10^−3^
	R^2^	0.018
Second-order kinetic	q_e_ (calc.) (mg g^−1^)	4.00
	K_2_ (mg g^−1^ min^−1^)	5.69
	R^2^	1
	q_e_ (exp.)	3.99
Interparticle diffusion	K_dif_ (min^1/2^)	0.0014
	C	0.60
	R^2^	0.615
Elovich	Β (mg g^−1^ min^−1^)	1.31
	α (g mg^−1^)	25,510.02
	R^2^	0.771

**Table 5:** Thermodynamic manners of adsorption of IV2R onto ZnO-NPs.

Temperature (°C)	∆G° (kJ mol^−1^)	∆H° (kJ mol^−1^)	ΔS° (J mol^−1^)
25	−5.22548	0.287	−0.018
30	−5.29248		
35	−5.3995		
45	−5.58672		
55	−5.76682		

**Table 6:** Comparison of the adsorption of organic dyes with other adsorbent materials.

Ref.	q_e_ (mg g^−1^)	Contact Time (min)	pH	Organic Dyes	Adsorptions
[66]	116.29	4		Ay 199	ZnO-NPs-AC
[19]	66.66	120	9	methylene blue	AC–ZnO
[67]	9.7	180	7	indigo carmine	Charcoal from rice bran
[68]	30	1200	-	indigo carmine	Charcoal from extracted residue of coffee beans
[69]	55.25	5	4	Titan yellow	Aloe vera
[70]	312.5	60–120	7.5	methylene blue	*Arthrospira platensis* biomass
[71]	2.13	480	4.9	tartrazine	Cellulose extracted from wheat residue
Current study	59.88	60–120	2	Ismate violet 2R (IV2R)	Green ZnO-NPs (prepared from red seaweed (*Pterocladia Capillacea*))

## Data Availability

The data that support the findings of this study are available from the authors upon reasonable request.
